# DCNet: Noise-Robust Convolutional Neural Networks for Degradation Classification on Ancient Documents

**DOI:** 10.3390/jimaging7070114

**Published:** 2021-07-12

**Authors:** Fitri Arnia, Khairun Saddami, Khairul Munadi

**Affiliations:** 1 Department of Electrical and Computer Engineering, Universitas Syiah Kuala, Banda Aceh 23111, Indonesia; f.arnia@unsyiah.ac.id (F.A.); khairul.munadi@unsyiah.ac.id (K.M.); 2 Telematics Research Center, Universitas Syiah Kuala, Banda Aceh 23111, Indonesia; 3 Ethnosciences Research Center, Universitas Syiah Kuala, Banda Aceh 23111, Indonesia

**Keywords:** degradation classification, document image analysis, deep learning, degraded ancient document images

## Abstract

Analysis of degraded ancient documents is challenging due to the severity and combination of degradation present in a single image. Ancient documents also suffer from additional noise during the digitalization process, particularly when digitalization is done using low-specification devices and/or under poor illumination conditions. The noises over the degraded ancient documents certainly cause a troublesome document analysis. In this paper, we propose a new noise-robust convolutional neural network (CNN) architecture for degradation classification of noisy ancient documents, which is called a degradation classification network (DCNet). DCNet was constructed based on the ResNet101, MobileNetV2, and ShuffleNet architectures. Furthermore, we propose a new self-transition layer following DCNet. We trained the DCNet using (1) noise-free document images and (2) heavy-noise (zero mean Gaussian noise (ZMGN) and speckle) document images. Then, we tested the resulted models with document images containing different levels of ZMGN and speckle noise. We compared our results to three CNN benchmarking architectures, namely MobileNet, ShuffleNet, and ResNet101. In general, the proposed architecture performed better than MobileNet, ShuffleNet, ResNet101, and conventional machine learning (support vector machine and random forest), particularly for documents with heavy noise.

## 1. Introduction

As one of the most important fields in image processing and pattern recognition, document image analysis helps machines or computers to understand a documented content. Unfortunately, analyzing ancient documents is quite challenging because of the severe degradation present in the documents. Accurate enhancement and restoration is crucial and must be implemented before analyzing ancient documents. An accurate enhancement would lead to a more readable and recognizable document. As a result, it is easier to restore and analyze the information inside the ancient document.

Current document analysis methods cannot tackle all types of degradation and only deal with a few issues. Additionally, the number and variations of datasets used to build a model cannot represent the real world [1]. Other problems arise when the document is digitalized using a device with low sensor sensitivity and/or under poor illumination conditions [2,3,4]. Here, the digitalization may generate additional, frequently heavy noise on the document. Inherently, most degraded ancient documents contain multiple degradation types within a single document [5] and additional digitalization noise, which can lead to the failure of simple image analysis.

Difficulties in enhancing ancient documents with multiple degradations can be overcome by partitioning the documents into several parts. These partitions create an easier mean to detect and classify the degradation type [6]. Recognition of degradation types in ancient documents simplifies the restoration and information-extraction steps. However, it is not feasible to recognize these types manually because of the massive number and variations of degraded ancient documents around the world. Hence, automatic degradation classification is required.

Currently, only a few works have reported automatic degradation classification on degraded document images. A work of automatic degradation classification on private datasets had been conducted using random forest [7]. Random forest as a traditional machine learning method uses spatial image information as features. The documents used in the experiment were synthetic images. The noise considered in the simulation was digitalization noise, such as border noise, skew, and rotation noises and degradation of back-to-front interference.

Shahkolei et al. had experimented with degradation classification using support vector machines (SVM) [8,9]. As another traditional machine learning technique, here, SVM was used to classify image quality based on a combination of spatial and frequency image features, namely Visual Document Quality Assessment Metrics (VDQAM) and Multi-distortion Document Quality Measure (MDQM). In this research, the degraded document image was classified into four types of degradation: paper translucency, stain, reader annotations, and worn holes. It was reported that a combination of MDQM and SVM achieved the best performance on detecting worn-hole distortion, with an average accuracy of 88.15%.

Performance of traditional machine learning in document classification over a large database is not favorable enough. Convolutional neural networks (CNN)—as a deep learning apporach—show superior performance on larger datasets [10]. Since AlexNet, which has a CNN architecture, won the ImageNet Large Scale Visual Recognition Challenge (ILSVRC) in 2012, CNN has performed remarkably in image classification and object recognition. The benchmarking CNN models, such as ResNet101 [11], MobileNetV2 [12], and ShuffleNet [13], have shown exceptional performances on classification of degradation types. Moreover, Saddami [14] presented performances of ResNet101, MobileNetV2, and ShuffleNet in classification of degradation types. However, to the best of our knowledge, there is no article, not even the newest review and survey articles [15,16] that has reported results on the capability of those models to classify degraded ancient documents in heavy-noise environments.

In this paper, we propose degradation classification methods for noisy degraded ancient documents using a deep neural network architecture, which is called document classification networks (DCNet). The proposed DCNet is designed to combat heavy noises inherent in low-quality document analysis application systems. We connected several stages of MobileNetV2 and ShuffleNet using an activation function called swish activation. Then, several ResNet blocks were attached and finally followed by the proposed transition layers.

We highlight three main contributions of this paper. First, we describe and reformulate degradation types in ancient documents. Second, we propose a novel CNN network design by modifying three benchmark CNN architectures, and a novel transition layer, for degradation classification on ancient documents, especially those with considerable noise. Third, in general, the proposed CNN performs better than ResNet, MobileNetV2, and ShuffleNet for documents with heavy noise, while the number of learning parameters of the proposed CNN is less than half of that of ResNet. This suggests that it can be embedded in a low-cost document image analysis device [17].

The rest of this paper is organized as follows: Section 2 presents a brief discussion of degradation types. Section 3 explains the proposed CNN architectures, while Section 4 presents the experimental setup. Section 5 discusses the result, and the conclusion is provided in Section 6.

## 2. Literature Review

In this section, we describe degradation in ancient documents, which were of interest to previous research studies, namely Ntirogiannis [18], Su [19], and Bataineh [20]. We describe four types of degradation caused by several factors: uniform degradation; bleed-through or show-through; faint text and low contrast; and smears, stains, or spots. We also present a brief introduction of support vector machine (SVM).

### 2.1. Degraded Ancient Documents

#### 2.1.1. Uniform Degradation

**Figure 1:**
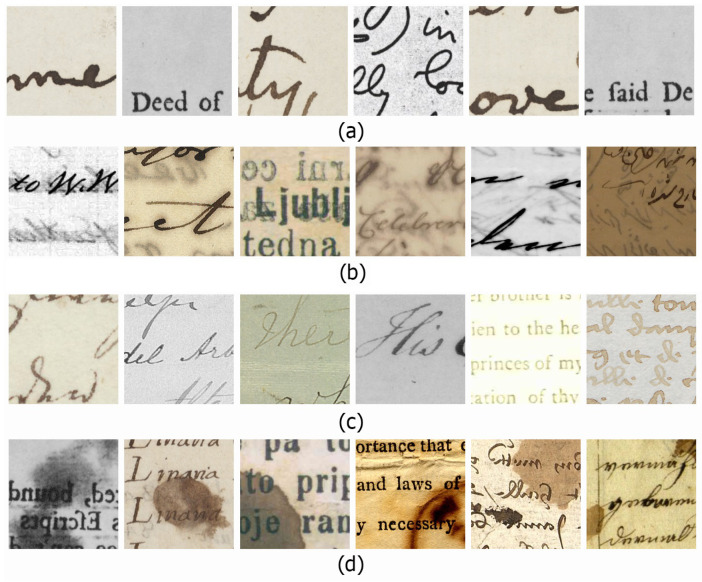
Examples of document images under different degradation [24,25,26,27,28,29,30,31,32]: (**a**) uniform degradation, (**b**) bleed-through-like degradation, (**c**) faint text and low contrast, (**d**) smear or stain or spot degradation.

If a document image contains degradation, especially in the background, but it is easy to read and extract the text, the degradation can be defined as a uniform degradation [21]. An ideally non degraded image has two histogram modalities: the foreground histogram and the background histogram. This is also the case for uniform degradation. The histogram is called a uniform or bimodal histogram [21]. A uniform degradation can be represented as in Equation (1)

(1) $$I_{dI}=I_{text}+I_{db}$$

where $I_{dI}$ is the degraded image, $I_{text}$ is the image of the main text, and $I_{db}$ is the degraded background. Figure 1a shows examples of uniform degradation.

Most ancient document images suffer from uniform degradation, and extracting text from a document with this degradation type is easier than other types of degradation. Using simple segmentation or binarization methods such as Otsu [22] or Sauvola [23] can be successful. According to Ntirogiannis [18], Otsu had a favorable performance on a document with a bimodal histogram. Consequently, a document that suffers from uniform degradation (has a bimodal histogram) should be successfully binarized by Otsu. In this research, uniform (UN) degradation is called UN degradation.

#### 2.1.2. Bleed-Through

A degradation on an ancient document that presents text from the other side of the documents is termed bleed-through, show-through, or back-to-front interference. Tonazzini [33] considered a bleed-through degradation as a combination of two images, in which the verso side experienced the transformation process. Each recto and verso of bleed-through document image can be formulated as in Equation (2) [33].

(2) $$\begin{array}{c} R_{x,y}=C_{11}I_{1}\left(x,y\right)+C_{12}I_{2}\left(x,y\right)\\ V_{x,y}=C_{21}I_{1}\left(x,y\right)+C_{22}I_{2}\left(x,y\right) \end{array}$$

where $R_{x,y}$ and $V_{x,y}$ are the recto and verso side of images, $C_{11}I_{1}\left(x,y\right)$ is the intensity of the main text or image in the recto side, $C_{12}I_{2}\left(x,y\right)$ is the defection of ink-bleed from the verso side, $C_{21}I_{1}\left(x,y\right)$ is the defection of ink-bleed from the verso to the recto side, and $C_{22}I_{2}\left(x,y\right)$ is the intensity of the verso side’s main text. Moreover, the bleed-through degradation can be simplified by summing up the uniform degraded image by ink from the verso side [34]. Based on Equation (1), the recto side can be formulated as in Equation (3)

(3) $$I_{rs}=I_{text}+I_{db}$$

where $I_{rs}$ is the recto-side image, $I_{text}$ is the main text, $I_{db}$ is the background of the image. As formulated by [34], the bleed-through is determined by Equation (4)

(4) $$I_{BT}=I_{rs}+I_{vs}$$

Therefore, the bleed-through degradation can be simplified as in Equation (5) [34]

(5) $$I_{BT}=I_{text}+I_{db}+I_{vs}$$

where $I_{BT}$ is the image with bleed-through or show-through degradation, $I_{text}$ is an image of the main text, $I_{db}$ is the degraded background of the image, and $I_{vs}$ is the image from the verso side. In this research, the bleed-through or show-through or back-to-front interference is called bleed-through (BLT) degradation. Examples of bleed-through degradation are shown in Figure 1b.

#### 2.1.3. Faint Text and Low Contrast

A faint-text image is a document image that suffers from faint or faded text. In this condition, the text can be damaged or missing. Moreover, this degradation results in a low-contrast image, in which the background and the text intensity are only slightly different. This type of degradation makes it difficult to distinguish the text from the background; therefore, it makes it be difficult to extract the text from this degradation.

According to Bataineh [20], low-contrast images have a low standard deviation value. This makes sense if the value is one of the parameters for depicting image contrast [35,36]. Furthermore, according to Lins [1], back-to-front interference or bleed-through degradation have a connection to faint text. Versa text of the degraded document can be a piece of document that suffered from faded or faint text. In this research, faint-text and low-contrast degradation is called Faint Text and Low Contrast (FTLC) degradation. Figure 1c shows examples of faint-text and low-contrast degradation.

#### 2.1.4. Smears, Stains, or Spots

Additional external factors that cover the text area of an ancient document image are labeled as smear, stain, or spot. Generally, the spot was labeled on degraded documents covered in small stains. In Figure 1d, we represent a smear, stain, or spot degradation as a summarization of a uniform document with an external factor covering the text. As mentioned in Equation (1), a uniform document was formulated as a sum of the main text and degraded background. Here, uniform degradation is covered by the external factor; hence, it is formulated as in Equation (6)

(6) $$I_{sss}=I_{text}+I_{db}+I_{ef}$$

where $I_{sss}$ is an image suffering from smear, stain, or spot degradation and $I_{ef}$ is an external factor that affected the document images. Basically, smear, stain, or spot degradation has commonalities with bleed-through; however, smears or stains are formed by an unknown object, while bleed-through represents the leakage of the versa text. In this research, the smear, stain, or spot degradation is called Smears, Stains, or Spots (SSS) degradation. Figure 1d shows examples of smear, stain, or spot degradation.

### 2.2. Support Vector Machine (SVM)

Support vector machine (SVM) is one of the best machine learning techniques for estimating nonlinear classification, regression, and multivariate functions [37]. Basic SVM operates as binary classifiers [38]. The SVM goal is to find the best hyperplane that separates two classes by maximizing margin distance between two outermost data points. The optimal SVM hyperplane is defined as in Equation (7)

(7) f(x)=wx+m

where f(x) is a hyperplane function, *w* and *m* are the weight and the bias of hyperplane, respectively. The optimal *w* and *m* can be computed by minimizing the distance between the two outermost data points. The distance is defined as in Equation (8)

(8) $$minimize(\frac{1}{2}{\left|W\right|}^{2}+C\sum_{i=1}^{N}\epsilon_{i})$$

where *C* is a trade-off penalty between margin and classification error and $\epsilon_{i}$ is an error control of hyperplane as in Equation (9) [39]

(9) $$y_{i}\left(wx+m\right)\geq 1-\epsilon_{i}$$

where $y_{i}$ is the class label which is defined as $y_{i}=\left\{+1,-1\right\}$.

To solve non-linear problems, the kernel approach, also known as the kernel trick, was proposed by [40]. The objective of the kernel trick is to map the input feature space into a high-dimensional space. Polynomial, Gaussian, and Radial Basis Function (RBF) are the most familiar kernel tricks used in SVM, while, based on the literature, RBF is the best RBF kernel trick due to its simplicity, efficiency, and adaptability. RBF is defined as in Equation (10) [41]

(10) $$K\left(x_{i},x_{j}\right)=exp(-\gamma ∥x_{i}-x_{j}{∥}^{2})$$

where γ is a regularization parameter, and $x_{i},x_{j}$ is features space from the input.

## 3. The Proposed Method

**Figure 2:**
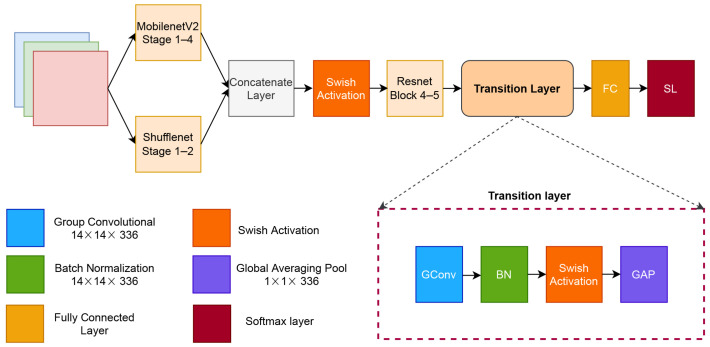
Architecture of the proposed model.

One approach to solving major problems in image classification is the use of deeper neural networks. A deeper network is composed of hundreds of layers and thousands of channels. In this section, we describe our proposed architecture for degradation classification on ancient document image, which is called document classification networks (DCNet) and is shown in Figure 2. Our proposed architecture is based on ResNet [11], MobileNetV2 [12], and ShuffleNet [13]. MobileNetV2 and ShuffleNet are widely considered to be fast and accurate CNNs for image classification, while ResNet is the most inspired and adapted CNN architecture [42,43].

First, the input image is trained in parallel using MobileNetV2 and ShuffleNet blocks. Furthermore, those blocks are concatenated and activated using the swish activation layer. The next stage is the ResNet block stage, followed by our proposed transition layer. Finally, the last layers are the softmax and classification layers.

ResNet is one of CNN benchmarking architectures, which proposes a residual layer for enhancing CNN performance on image classification and object detection [11]. The residual layer of the ResNet is formulated as in Equation (11)

**Figure 3:**
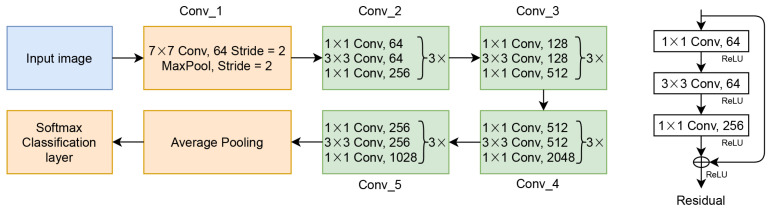
The basic architecture of ResNet.

(11) Y=f(x)+x

where *Y* is the output of the residual layer, *x* is the input from the previous layer, and f(x) is residual mapping from the previous layer. Based on Equation (11), *Y* is the result of the element-wise addition layer of *x* after being trained with some computation processes with *x*. Figure 3 shows ResNet architecture.

**Figure 4:**
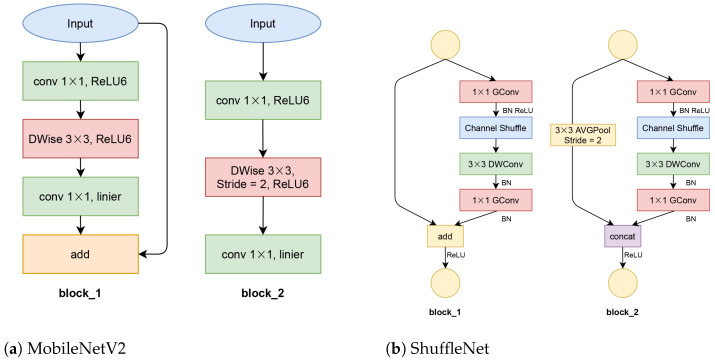
The basic architecture of MobileNetV2 and ShuffleNet.

MobileNetV2 is a fast, mobile-based CNN, which was extended from MobileNet [44]. MobileNetV2 proposed a new bottleneck of depthwise separable convolution and residual inverted blocks. It also modified the rectified linear units (ReLU) activation layer by limiting the positive value to six; this ReLU version is called ReLU6. However, the reason for limiting was never mentioned in MobileNet [44] or MobileNetV2. Basically, MobileNetV2 consists of two block structures based on stride numbers, which is shown in Figure 4a. Our proposed networks are arranged in four blocks_1 and two blocks_2. This composition is half of the original MobileNetV2.

ShuffleNet proposed point-wise group convolutions and channel shuffling methods to cross features from different channels of CNN. This arrangement resulted in an efficient CNN, especially for mobile application. ShuffleNet consists of two main stages as shown in Figure 4b. In our proposed network, we used only one block_1 and one block_2. This structure has approximately half of the original ShuffleNet.

After concatenating MobileNetV2 and ShuffleNet, the weight value is activated using swish activation layers. Introduced by the Google Brain team, Swish is a self-gated activation function that is considered better than ReLU activation [45]. Swish is formulated as in Equation (12) [46].

(12) SwishX=x×sigmoid(β,x)

where β is either a constant or a trainable parameter. Unlike the ReLU function, which is a monotonic and rigid curve, Swish has a non-monotonic and smooth curve. In our proposed network, we set the β value to be 1. Therefore, we have a simple sigmoid parameter on the Swish activation, which resulted in simple computation. Our transition layer consists of a group convolution layer, a batch normalization layer, a swish layer, a global averaging pooling (GAP), and a fully connected layer. The transition layers end in a softmax layer.

The group convolution layer performs the convolution process by separating the channel into several groups. In the proposed network, the group convolution layer has 336 groups with a filter of 5×5 pixels. Group convolution has several advantages over the conventional convolutional layer. It is more efficient in training, and the model has fewer parameters. The group convolution layer is denoted as in Equation (13) [47].

(13) $$X_{k+1}=X_{k}⊗W_{k}$$

$X_{k}$ denotes input feature maps of the k layer, $W_{k}$ represents the filter of the k layer, and ⊗ is the convolution operator. $X_{k}$ is represented as $Xk=\{x_{k1},x_{k2},\ldots ,x_{kG}\}$, and G is the number of groups of $X_{k}$. $W_{k}$ is denoted as $Wk=\{w_{k1},w_{k2},\ldots ,w_{kG}\}$. Hence, the group convolution layer is formulated as in Equation (14)

(14) $$X_{k+1}=\left\{W_{k1}*X_{k1},W_{k2}*X_{k2},\ldots ,W_{kG}*X_{kG}\right\}$$

Batch normalization (BN) was proposed for solving complicated network training due to the inconsistency of the layer’s weighted distribution of each iteration during the training progress [48]. BN offers a normalization process to keep a fixed mean and variance of the layers. BN resulted in many advantages, such as accelerating the training progress by enabling high learning rates and saving the training networks from saturated modes. BN also works as a regularization, so it can replace the dropout stage. A BN is formulated as in Equation (15)

(15) $${\hat{x}}^{\left(k\right)}=\frac{x^{\left(k\right)}-E\left[x^{\left(k\right)}\right]}{\sqrt{var\left[x^{\left(k\right)}\right]}}$$

where ${\hat{x}}^{\left(k\right)}$ is the normalized feature value, $x^{\left(k\right)}$ is the input from previous layer, and $E[x^{\left(k\right)}]$ and $Var[x^{\left(k\right)}]$ are the expectation and variance over the training dataset.

Furthermore, GAP is applied to reduce the features of the previous layer by using the average pooling approach. The idea of GAP is to obtain an activation map for every category of the classification tasks [49]. GAP calculates one value using average pooling for every channel of the feature.

## 4. Experimental Setup

### 4.1. Datasets

**Figure 5:**
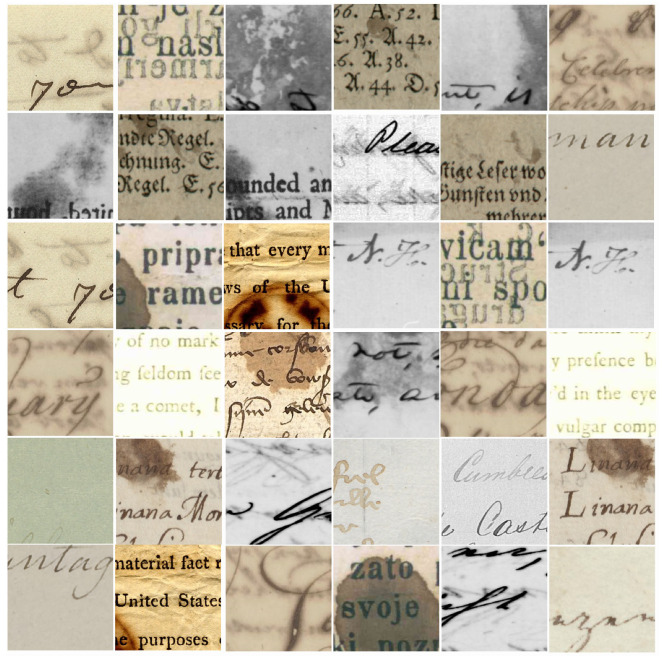
Example of image patches used for data training [24,25,26,27,28,29,30,31,32].

We generated 30,000 image patches from 10 public and 1 private dataset, with a size of 224×224 pixels. The degraded images were obtained from Document Image Binarization Contest (DIBCO) 2009–2018 [24,25,26,27,28,29,30,31], Persian Heritage Image Binarization Dataset (PHIBD) [32], and the private Jawi dataset [50,51]. From these datasets, we identified four categories of image degradation: uniform; bleed-through; faint test and low contrast; and smears, stains, and spots. We collected 7500 image patches from each categories, which made a dataset with 30,000 image patches in total. The dataset was grouped into training, validation, and testing parts with a composition of 80%:10%:10%. Hence, we had 24,000 images for training, 3000 images for validation, and 3000 images for testing. Figure 5 shows examples of image patches for training the models.

Image patches in our database were created by selecting images from the original dataset containing the degradation types mentioned above. For each degradation category, we chose degradation that has been confirmed/classified in the previous literature. Image parts that did not contain noise were ignored. We took patches with size of 224×224 pixels, without doing any pre-processing. If a region had been patched, then the next region taken as a patch was an area least 50 pixels away from the previous patch, so that there would be no overlapping patch.

### 4.2. Parameter Settings

For training purposes, we used adaptive moment estimation, or Adam, as the optimizer [52]. Adam is an adaptive learning gradient with momentum and magnitude of the gradient. Adam is proposed for improving RMSprop as an adaptive learning rate for bias correction [53]. The square gradient decay factor that was used for the Adam optimizer is 0.999 and was the default value used in the paper. The initial learning rate of the training process was set to $10^{-3}$. We used cross-entropy as the loss function. Furthermore, we used L2Regularization with the value of lambda (λ) equal to $10^{-5}$. These parameters were obtained as the best values based on our hyper-parameter experiments. Due to resource availability, we trained each CNN model using 25,000 batches size.

### 4.3. Simulations

**Figure 6:**
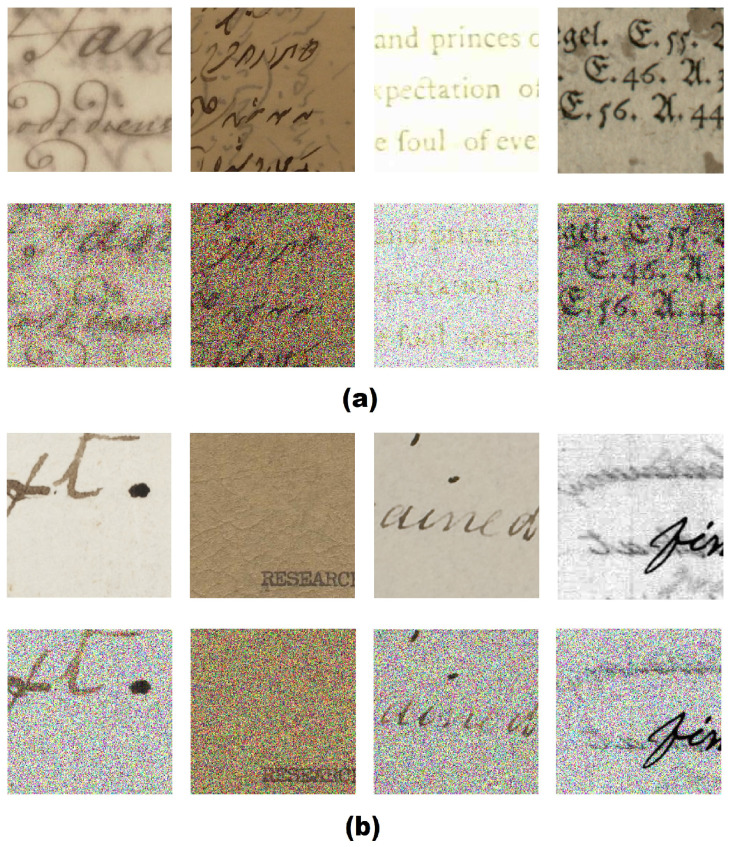
Example of images with heavy noise [24,25,26,27,28,29,30,31,32], where: (**a**) the document image suffered from zero-mean Gaussian noise (bottom), and (**b**) the document image suffered from speckle noise (bottom).

To evaluate the robustness of our proposed model, we trained DCNet and three CNN benchmarking architectures, namely MobileNet, ShuffleNet, and ResNet101, for comparison purposes. The training was performed on (1) noise-free images and (2) heavy-noise images. The heavy-noise images consisted of heavy zero-mean Gaussian noise (ZMGN)-noise images (σ = 0.125) and heavy speckle-noise images (σ = 0.25). Thus, we obtained three models from each architecture, which made a total of 12 models. Then, we tested each of the models with the ZMGN and speckle noise images with different levels of noise (different σ). We tested our model on ZMGN by adjusting variance (σ) to σ = {0.005,0.01,0.05,0.1,0.125}, and, for speckle noise, we adjusted variance (σ) to σ = {0.05,0.075,0.1,0.15,0.2,0.25}. Figure 6 shows examples of the testing images after applying various noises.

Furthermore, we compared DCNet with traditional machine learning, namely support vector machine (SVM) and random forest (RF) [7,9]. We set SVM kernel to RBF function and trained it using one-versus-one approach. We used 1000 trees to perform the RF training process. We used the visual document quality assessment metric (VDQAM) method as the feature extractor [8].

To show the robustness of our proposed method in classifying degradation types, we present classification results of the four degradation types, using the f-measure (FM) metric. In this case, we trained the model with noise-free images.

### 4.4. Evaluation Performance

We evaluated our proposed model using accuracy and F-measure *(FM)*. Accuracy is formulated as in Equation (16)

(16) $$Accuracy=\frac{TP+TN}{TP+FP+TN+FN}$$

where true positive *(TP)* is the correct prediction of the class image, true negative *(TN)* is the correct prediction of the different class, false positive *(FP)* is an image that is wrongly predicted as the class image, and false negative *(FN)* is a class image that is wrongly predicted as a different class.

Furthermore, F-measure *(FM)* is is determined by Equation (17)

(17) $$FM=\frac{2\times Recall\times Precision}{Recall+Precision}$$

where recall is presented as $\frac{TP}{TP+FN}$ and precision is $\frac{TP}{TP+FP}$. In the subsequent parts we show the training and validation loss graphics and testing accuracy.

## 5. Result and Discussion

### 5.1. Training Results

**Figure 7:**
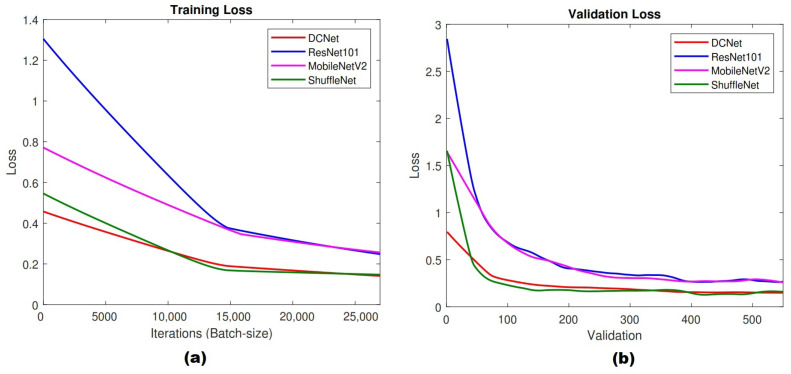
Training performance: (**a**) training loss, (**b**) validation loss.

Figure 7 shows training and validation loss charts of all CNN architectures. Figure 7a presents the overall training loss of training progress, while Figure 7b shows validation loss. DCNet and ShuffleNet obtained the lowest loss value during the training process, while ResNet101 and MobileNetv2 achieved the highest loss value. This result indicated that DCNet has a good performance during training process. Furthermore, the validation loss chart shows corresponding trends with the training loss. Based on Figure 7b, DCNet accomplished validation loss under 0.5 starting from 100 validations, while ResNet101 and MobileNetV2 obtained similar performance after 200 validations. ShuffleNet showed a similar validation loss with DCNet, but it was quite unstable (shown by fluctuated graphs). The proposed model resulted in a more stable performance compared with ResNet101, MobileNetV2, and ShuffleNet.

### 5.2. Results of the Noise-Free Model

**Figure 8:**
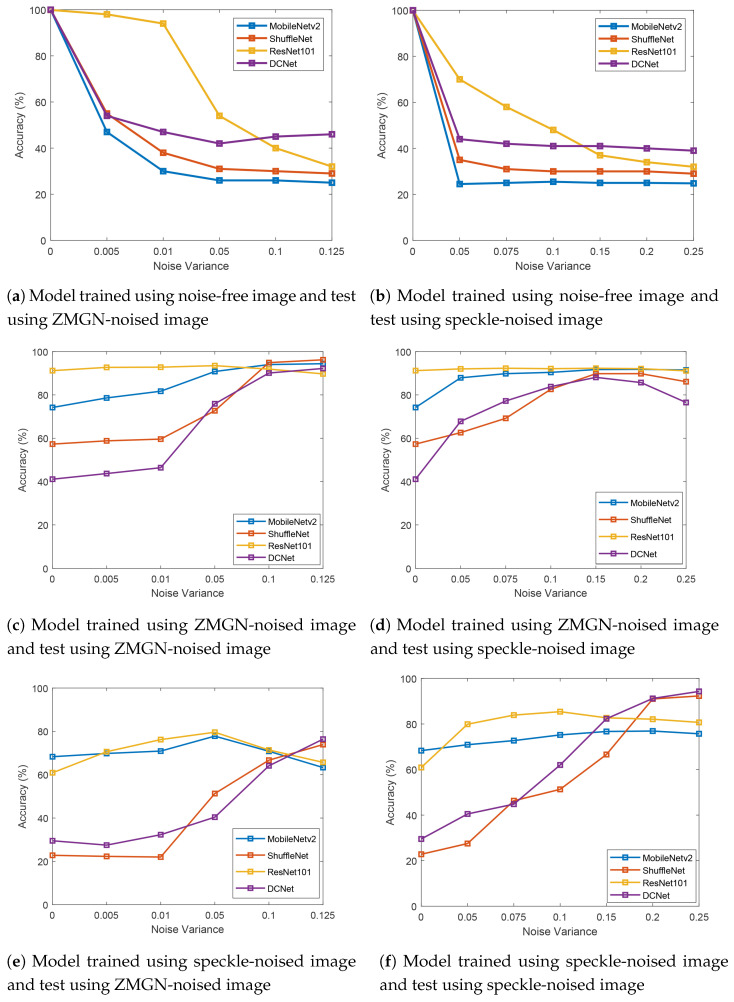
Testing results on different convolutional neural network (CNN) models and noises.

In the first simulation condition, the CNN architectures were trained on noise-free images and tested on images with zero-mean Gaussian noise (ZMGN) and speckle noise. The testing and comparison results are shown in Figure 8a,b.

Based on Figure 8a, ResNet101’s accuracy reached almost 100% when the noise level was low (σ=0.005). Moreover, DCNet, MobileNetV2, and ShuffleNet obtained accuracy between 40% and 60%. However, when the noise level was increased, ResNet101’s performance dropped drastically to around 40%; in contrast, the accuracy of DCNet, MobileNetV2, and ShuffleNet decreased only by around 8%, 8%, and 5%, respectively. DCNet achieved better performance when σ≥ 0.1, which indicated heavier noise.

A similar trend was also present with speckle noise (see Figure 8b), DCNet achieved higher accuracy when the noise level was high (σ> 0.1). Under heavy noise, the proposed model achieved accuracy between 35 and 45%, while ResNet101, MobileNetV2, and ShuffleNet only obtained accuracy between 33 and 37%, 24 and 26%, and 29 and 31%, respectively. Under light noise (σ< 0.1), ResNet101 performed the best, while DCNet came in second place. DCNet resulted in a noise-robust performance and the best classification result against heavy noise levels, compared with ResNet101, MobileNetV2, and Shufflenet. A heavier noise level reduced ResNet101’s performance; the chart experienced a gradual decline, but ResNet101 showed a promising classification performance on light ZMGN and speckle noise. MobileNetV2 and ShuffleNet showed a lower accuracy in both noise conditions.

### 5.3. Results of the ZMGN-Noise Model

In the second simulation condition, CNN models were trained on heavy zero-mean Gaussian noise (ZMGN) with noise variance σ= 0.125 and tested on zero-mean Gaussian noise (ZMGN) and speckle noise with different noise levels. The testing and comparison results are shown in Figure 8c,d. Based on Figure 8c, DCNet shows a promising performance shown by an increasing accuracy values starting from noise variance (σ) 0.01. DCNet accuracy is higher than that of ResNet101 when σ = 0.125.

In the training stage, the images were trained with heavy ZMGN noise images (σ = 0.125). This may explain why, as noise level increased in the testing stage, the the performance of all CNN models improved. At σ = 0.125, each model achieved its best accuracy, and all models performed similarly.

Based on Figure 8d, DCNet and ShuffleNet show a moderate improvement. However, the accuracy of both DCNet and ShuffleNet declined from σ=0.2. MobileNetV2 and ResNet101 remained constant even when the noise variance was increased. Here, ResNet101 achieved the most stable performance when the CNN was trained on ZMGN noise.

### 5.4. Results of the Speckle-Noise Model

In the third simulation condition, CNN models were trained on heavy speckle noise with noise variance (σ) of 0.25 and tested on zero-mean Gaussian noise (ZMGN) and speckle noise with different noise levels. The testing and comparison results are shown in Figure 8e,f. Based on Figure 8e, DCNet demonstrates a promising performance with a significant increment of accuracy values starting from noise variance σ=0.01 and reached the best performance when noise variance σ=0.25. ShuffleNet showed a a similar tendency with the DCNet, while ResNet101 and MobileNetv2 showed a fluctuated performance. Figure 8f presents testing results of degradation classification that applied speckle noise on the testing image. Similar to what is shown in Figure 8e, DCNet resulted in a promising performance on degradation classification, particularly on heavy noise images. DCNets graph showed a significant improvement in accuracy values, from the smallest to the biggest noise levels. Its best performance was achieved at the noise variance σ=0.25. ShuffleNet shows a similar performance to DCNet, while ResNet101 and MobileNetV2 show a stable performance with slight accuracy increment.

### 5.5. Comparison with Traditional Machine Learning

Table 1 and Table 2 show comparison performance of DCNet, as a deep learning approach, with traditional machine learning namely support vector machine (SVM) and random forest (RF). Based on Table 1, DCNet resulted in the best performance in classifying degradation on ancient documents with noise-free training images. The heavier noise only slightly affects the SVM and RF performance. The accuracy values of classification were similar, for either SVM or RF, when the DCNet trained with ZMGN and speckle noise images. The accuracy of SVM and RF was lower than 42% if ZMGN was applied on the testing images. In contrast, DCNet reached an accuracy of 92%. In general, a better result was achieved when the testing noise was speckle noise, as shown in Table 2. The SVM and RF can reach an accuracy of 50%, while DCNet achieved an accuracy of 94%, which was significantly higher than those of SVM and RF.

The results of SVM and RF tend to be similar even if various noise variance was added to the image. In SVM and RF, we need to extract the hand-crafted feature, Here, we used the visual document quality assessment metric (VDQAM) method as the feature extractor. We observed that varying noise variance did not change the resulted VDQAM features; thus, it would affect the classification performance. In other words, when we use traditional machine learning in classification, it was proven that the classification was determined by the hand-crafted feature, not by the visual image condition. According to Table 1 and Table 2, applying ZMGN noise on testing images was successfully handled by the DCNet that was trained with noise-free images and noisy images. For traditional machine learning, the model that was trained using speckle images has a better performance when tested either on ZMGN or speckle noise images. These results indicated that the DCNet has a better performance as compared to traditional machine learning (in this case SVM and RF) in degradation classification tasks of noisy images.

### 5.6. Performance of Deep Learning Models in Degradation Classification

In this subsection, we present the performance of CNN models in classifying degradation types. We did the training on noise-free images. Table 3 and Table 4 show the performance of CNN models in classifying degradation types on ancient document images.

Table 3 presents a comparison of CNNs’ model performance on ZMGN. DCNet achieved the best performance in classifying FTLC and SSS degradations with heavy noise (σ> 0.05). DCNet also accomplished a similar performance to MobileNetV2 on BLT degradation. Unfortunately, the proposed model did not succeed in recognizing Uniform (UN) degradation. Under the same condition, the MobileNetV2 obtained a better performance UN, which was shown by a non-zero values. In contrast, under light noise conditions, ResNet101, which has a much larger model than the others, obtained the highest FM result, followed by DCNet, MobileNetV2, and ShuffleNet. In general, the BLT and the FTLC are easier to classify compared with the SSS and the UN. During the experiment, we found that most of the SSS- and UN-degraded images were classified as BLT or FTLC.

Table 4 presents a comparison of CNNs’ model performance on documents with speckle noise. DCNet achieved the best performance in classifying FTLC and SSS degradations under heavy noise conditions (σ> 0.1). It accomplished a similar performance to MobileNetV2 on BLT degradation. Similar to ZMGN noise, the proposed model did not succeed in recognizing UN degradation. MobileNetV2 obtained a better performance on UN degradation (it has non-zero values). However, it failed to classify document images with FTLC and SSS degradation, as well as ShuffleNet. Under light noise conditions (σ< 0.1), ResNet101 obtained the best result for all noise types.

According to Table 3 and Table 4, ResNet101 showed acceptable performance only on light noise images, but its performance dropped dramatically on heavy noise of all noise types. Under heavy noise, MobileNetV2 and ShuffleNet failed in classifying noisy images with FTLC and SSS degradations; these models had worse performances than DCNet. As for UN, MobileNetV2, ShuffleNet, DCNet, and ResNet101 obtained almost zero FM values for all noise conditions. It was determined that all models failed in classifying UN with any noise conditions. Therefore, it can be inferred that MobileNetV2 and ShuffleNet works only on BLT degradation.

ResNet101 has the limitation of being implemented in a low-cost system because it only works on light-noise images and fails on heavy-noise images. MobileNetV2 and ShuffleNet are also inappropriate for implementation in a low-cost document analysis application because they only performed well on BLT degradation. The facts that a low-cost digitalization device resulted in heavy-noise images, and that the simulations showed that DCNet is robust to heavy noises, confirm that the proposed architecture is the suitable CNN for a low-cost document image analysis system.

DCNet consists of half of ResNet101, MobileNet, and ShuffleNet and self-proposed transition layers. Table 5 shows the number of learning parameters of all the networks. DCNet has 18.9 million parameters, which is fewer than ResNet101 but more than MobileNetV2 and ShuffleNet. However, MobileNetV2 and ShuffleNet showed a less stable performance compared with DCNet for all conditions. Therefore, we argue that increasing learning parameters in DCNet is a compromise to accomplish robust degradation classification on documents with heavy noise.

## 6. Conclusions

We propose a novel CNN architecture for degradation-type classification of noisy ancient documents. The proposed model is called degradation classification network (DCNet). DCNet is a combination of MobileNetV2, ShuffleNet, ResNet101, with newly proposed transition layers. The degradation types under consideration were bleed-through; faint text and low contrast; smears, stains, or spots; and uniform degradations. We trained the DCNet using (1) noise-free document images and (2) heavy-noise document images in which the noise was zero mean Gaussian noise (ZMGN) and speckle noise. Then, we tested the resulting models with document images containing the ZMGN and speckle noise images with different noise levels. We compared the performance of DCNet with three CNN benchmarking architectures, namely MobileNet, ShuffleNet, and ResNet101, in terms of training loss, validation loss, and accuracy. We also extended our experiments to assess two machine learning approaches’ (support vector machine and random forest) classification performance. It turned out that the DCNet demonstrated a better performance as compared to other methods, particularly for documents with heavy noise.

## Figures and Tables

**Table 1:** Comparison of accuracy value with traditional machine learning on zero mean Gaussian noise (ZMGN) testing images. Bold fonts indicate the best results.

Testing Image Applied ZMGN Noise						
Model Trained on noise-free image						
Methods	σ = 0.0	σ = 0.005	σ = 0.01	σ = 0.05	σ = 0.1	σ = 0.125
DCNet	**100.0**	**54.00**	**47.0**	**42.00**	**45.00**	**46.00**
SVM	78.00	35.60	35.00	35.57	35.77	34.03
RF	70.17	27.17	27.03	27.17	26.57	28.67
Model Trained on ZMGN image						
Methods	σ = 0.0	σ = 0.005	σ = 0.01	σ = 0.05	σ = 0.1	σ = 0.125
DCNet	**41.10**	**43.70**	**64.40**	**75.90**	**90.10**	**92.20**
SVM	25.00	25.23	25.53	23.70	21.57	22.33
RF	37.37	34.32	34.13	37.70	41.33	41.17
Model Trained on Speckle image						
Methods	σ = 0.0	σ = 0.005	σ = 0.01	σ = 0.05	σ = 0.1	σ = 0.125
DCNet	29.50	27.50	32.30	**40.40**	**64.10**	**76.40**
SVM	25.00	31.73	31.67	30.63	30.23	31.50
RF	**64.97**	**34.17**	**34.50**	36.70	36.47	35.80

**Table 2:** Comparison of accuracy value with traditional machine learning on speckle testing image. Bold fonts indicate the best results.

Testing Image Applied Speckle Noise							
Model Trained on noise-free image							
Methods	σ = 0.0	σ = 0.05	σ = 0.075	σ = 0.1	σ = 0.15	σ = 0.2	σ = 0.25
DCNet	**100.0**	**44.00**	**42.00**	**41.00**	41.00	40.10	39.00
SVM	78.00	40.13	40.50	34.03	**44.90**	**45.47**	**46.03**
RF	70.17	23.57	23.83	28.67	24.37	23.47	23.67
Model Trained on ZMGN image							
Methods	σ = 0.0	σ = 0.05	σ = 0.075	σ = 0.1	σ = 0.15	σ = 0.2	σ = 0.25
DCNet	**41.1**	**67.8**	**77.2**	**83.8**	**88.1**	**85.7**	**76.5**
SVM	25.00	31.03	34.57	37.47	40.37	43.90	46.37
RF	37.37	42.40	40.90	41.83	48.63	53.05	55.83
Model Trained on Speckle image							
Methods	σ = 0.0	σ = 0.05	σ = 0.075	σ = 0.1	σ = 0.15	σ = 0.2	σ = 0.25
DCNet	29.5	40.5	44.8	**62.0**	**82.3**	**91.2**	**94.3**
SVM	25.00	40.93	46.27	53.60	66.17	71.63	72.63
RF	**65.37**	**44.87**	**50.80**	56.80	65.97	73.50	74.67

**Table 3:** Performance of convolutional neural network (CNN) models on different types of degradation and additional zero-mean Gaussian noise (in F-measure). Bold fonts indicate the best results.

σ = **0.005**				
	**BLT (%)**	**FTLC (%)**	**SSS (%)**	**UN (%)**
ResNet101	**98.14**	**99.10**	**98.22**	**97.19**
MobileNetV2	48.60	79.41	27.11	1.73
ShuffleNet	50.89	43.26	79.45	49.87
DCNet	59.26	68.00	55.49	0.00
σ = **0.01**				
	**BLT (%)**	**FTLC (%)**	**SSS (%)**	**UN (%)**
ResNet101	**93.36**	**96.05**	**91.83**	**93.96**
MobileNetV2	45.20	25.75	10.79	0.00
ShuffleNet	44.33	11.32	62.35	13.11
DCNet	49.34	59.85	47.44	0.00
σ = **0.05**				
	**BLT (%)**	**FTLC (%)**	**SSS (%)**	**UN (%)**
ResNet101	**67.73**	**52.51**	**44.12**	**1.78**
MobileNetV2	44.72	0.00	0.20	0.38
ShuffleNet	41.65	4.76	25.02	0.00
DCNet	32.85	51.94	42.89	0.00
σ = **0.1**				
	**BLT (%)**	**FTLC (%)**	**SSS (%)**	**UN (%)**
ResNet101	33.78	43.51	25.63	0.00
MobileNetV2	**45.67**	0.00	0.00	0.00
ShuffleNet	40.89	2.18	0.00	**0.51**
DCNet	36.65	**56.37**	**40.82**	0.00
σ = **0.125**				
	**BLT (%)**	**FTLC (%)**	**SSS (%)**	**UN (%)**
ResNet101	27.14	42.52	19.98	0.00
MobileNetV2	**45.87**	0.00	0.00	**0.48**
ShuffleNet	40.67	0.00	0.00	0.00
DCNet	40.58	**58.15**	**41.53**	0.00

**Table 4:** Performance of CNN models on different types of degradation and additional speckle noise (in F-measure). Bold fonts indicate the best results.

σ = **0.05**				
	**BLT (%)**	**FTLC (%)**	**SSS (%)**	**UN (%)**
ResNet101	**87.57**	**66.82**	**83.72**	**35.02**
MobileNetV2	44.96	0.00	6.00	1.42
ShuffleNet	43.11	6.71	47.68	2.37
DCNet	43.42	55.48	45.56	0.00
σ = **0.075**				
	**BLT (%)**	**FTLC (%)**	**SSS (%)**	**UN (%)**
ResNet101	**77.34**	**57.85**	**65.32**	**11.08**
MobileNetV2	44.84	0.00	4.85	1.07
ShuffleNet	42.02	6.63	30.94	0.00
DCNet	39.54	55.80	45.20	0.00
σ = **0.1**				
	**BLT (%)**	**FTLC (%)**	**SSS (%)**	**UN (%)**
ResNet101	**62.12**	51.85	**50.75**	**2.91**
MobileNetV2	44.95	0.00	2.54	0.53
ShuffleNet	41.40	5.57	21.91	0.00
DCNet	37.74	**55.82**	44.60	0.00
σ = **0.15**				
	**BLT (%)**	**FTLC (%)**	**SSS (%)**	**UN (%)**
ResNet101	37.23	45.4	27.73	0.00
MobileNetV2	**45.32**	0	1.76	**0.64**
ShuffleNet	41.14	6.39	18.68	0.00
DCNet	38.06	**59.82**	**42.27**	0.00
σ = **0.2**				
	**BLT (%)**	**FTLC (%)**	**SSS (%)**	**UN (%)**
ResNet101	33.57	43.87	20.14	0
MobileNetV2	**45.28**	0	0.59	**0.64**
ShuffleNet	41.14	6.2	18.84	0.00
DCNet	39.63	**58.13**	**36.03**	0.00
σ = **0.25**				
	**BLT (%)**	**FTLC (%)**	**SSS (%)**	**UN (%)**
ResNet101	31.89	42.89	17.51	0
MobileNetV2	**45.32**	0	0	**0.64**
ShuffleNet	41.02	6.19	17.51	0
DCNet	42.38	**47.26**	**26.09**	0

**Table 5:** Comparison of learning parameters with baseline CNN models.

Models	Number of Parameters (millions)
ResNet101	44.6
MobileNetV2	3.5
ShuffleNet	1.4
DCNet	18.9

## Data Availability

The study did not report any data.
